# Association between estimated glucose disposal rate and testosterone level in US adult men: insights from NHANES 2013-2016

**DOI:** 10.1093/sexmed/qfaf075

**Published:** 2025-09-15

**Authors:** Hege Bian, Yuzhong Zhang, Kun Liu

**Affiliations:** Supply Chain Department, Hefei BOE Hospital, Hefei, 230013, China; Supply Chain Department, Hefei BOE Hospital, Hefei, 230013, China; Department of Surgery, The Third Affiliated Hospital of Anhui Medical University (The First People’s Hospital of Hefei), Hefei, 230061, China

**Keywords:** estimated glucose disposal rate, total testosterone, testosterone deficiency, insulin resistance, NHANES

## Abstract

**Background:**

Emerging evidence suggests that insulin sensitivity plays a role in testosterone regulation. The estimated glucose disposal rate (eGDR) is a validated metabolic marker reflecting insulin resistance (IR). However, the relationship between eGDR and testosterone levels in adult men remains unclear.

**Aim:**

This study aimed to examine the association between eGDR, total testosterone (TT) levels, and testosterone deficiency (TD) risk.

**Methods:**

Data from the 2013-2016 National Health and Nutrition Examination Survey (NHANES) were analyzed. Weighted multivariable linear and logistic regression models were used to evaluate the association between eGDR, TT levels, and TD risk (TT <300 ng/dL). A smoothing spline curve fitting approach was applied to assess the shape of the relationship. Subgroup analyses and interaction tests were conducted to explore potential effect modifications. Receiver operating characteristic (ROC) analysis was performed to assess the predictive ability of eGDR for TD.

**Outcomes:**

eGDR was calculated using waist circumference (WC), hypertension (HTN), and glycated hemoglobin (HbA1c).

**Results:**

A total of 4087 male participants were included in the final analysis. After adjusting for all covariates, higher eGDR was significantly associated with increased TT levels (*β* = 31.83, 95% CI, 22.13-41.54, *P* < .001) and a lower risk of TD (OR = 0.68, 95% CI, 0.58-0.80, *P* = .002). Quartile analysis showed that participants in the highest eGDR quartile (Q4) had significantly higher TT levels than those in Q1 (*β* = 147.27, 95% CI, 66.99-227.55, *P* = .02) and a markedly reduced TD risk (OR = 0.20, 95% CI, 0.06-0.70, *P* = .03). Smoothing spline curve fitting approach confirmed a linear relationship between eGDR and TT levels, as well as an inverse association with TD risk. A significant interaction was observed for diabetes status (*P* for interaction = .001), indicating a potential modifying effect. ROC analysis demonstrated that eGDR had moderate predictive ability for TD (AUC = 0.6839, 95% CI, 0.6659-0.7019).

**Clinical Implications:**

eGDR may serve as a useful metabolic marker for identifying individuals at risk of TD.

**Strengths and Limitations:**

GDR may serve as a valuable metabolic marker for identifying individuals at risk of TD; due to its cross-sectional design, we cannot establish causality between eGDR and testosterone levels.

**Conclusion:**

These findings suggest that eGDR is associated with testosterone levels and TD risk in adult men, highlighting the potential metabolic link between insulin sensitivity and testosterone regulation.

## Introduction

Testosterone, a steroid hormone primarily secreted by Leydig cells and regulated by the hypothalamic-pituitary-gonadal (HPG) axis, plays a central role in the development and maintenance of male secondary sexual characteristics.^1–3^ Additionally, testosterone levels are closely associated with cardiovascular function, cognitive ability, muscle mass, bone mineral density, and erythropoiesis.^4^ testosterone deficiency (TD), defined as a total serum testosterone level below 300 ng/dL, affects approximately 30% of men aged 40-79 years.^5^^,^^6^ Beyond sexual dysfunction, low testosterone levels are linked to an increased risk of osteoporosis, diabetes, metabolic syndrome, and cognitive decline, as well as higher cardiovascular and all-cause mortality.^7–10^ Given its detrimental effects on multiple organ systems and significant impact on quality of life, TD has emerged as a major public health concern.

Metabolic factors, including obesity, hyperlipidemia, hypertension (HTN), and insulin resistance (IR), are recognized risk factors for TD.^10^ Among them, IR is strongly associated with low testosterone levels, as it impairs Leydig cell function and reduces testosterone secretion.^11^ Notably, emerging evidence suggests a bidirectional relationship: metabolic disturbances may suppress testosterone production, while low testosterone levels may in turn exacerbate metabolic dysfunction. This interplay underscores the importance of identifying metabolic indicators that may predict or reflect TD, particularly in the context of systemic metabolic health. The hyperinsulinemic-euglycemic clamp is considered the gold standard for assessing insulin sensitivity, providing the most accurate measurement of IR.^12^^,^^13^ However, its invasiveness and high cost limit its feasibility in clinical and epidemiological settings. As alternative methods, the homeostatic model assessment of insulin resistance (HOMA-IR)^14^ and the triglyceride-glucose (TyG) index^15^ are commonly used for IR screening. However, HOMA-IR, which is based on fasting glucose and insulin levels, may be influenced by exogenous insulin administration, particularly in diabetes mellitus (DM) individuals, potentially reducing its accuracy in certain populations.^14^ The TyG index has been linked to metabolic syndrome (MetS) and its outcomes but may not fully capture the complexity of IR, especially when considering key MetS components such as central obesity and HTN.^16^^,^^17^

In this context, the estimated glucose disposal rate (eGDR) has emerged as a promising tool for assessing IR. Calculated using waist circumference (WC), HTN, and glycated hemoglobin A1c (HbA1c), eGDR provides a more comprehensive measure of insulin sensitivity. Initially developed for evaluating IR in patients with type 1 diabetes mellitus (T1DM),^18^ eGDR has since been associated with stroke, diabetic nephropathy, and mortality in individuals with type 2 diabetes mellitus (T2DM).^19^^,^^20^ Recently, eGDR has been recognized for its strong correlation with MetS and its predictive value for all-cause and cardiovascular disease (CVD) mortality. Compared to TyG and HOMA-IR, eGDR demonstrated superior predictive ability for all-cause mortality, underscoring its potential as a valuable clinical risk assessment tool.^21^ By incorporating WC, HbA1c, and HTN, eGDR enables a comprehensive evaluation of insulin sensitivity without the need for direct insulin measurements.

Although IR is known to influence testosterone levels, most studies have focused on traditional IR markers such as HOMA-IR and the TyG index, both of which have notable limitations. The eGDR, a noninsulin-based surrogate incorporating WC, HTN, and HbA1c, provides a broader assessment of metabolic dysfunction. However, its association with testosterone levels in adult men remains unexplored in population-based studies. Clarifying this relationship may offer new insights into the metabolic determinants of TD. Furthermore, current biomarkers of TD often rely solely on serum testosterone levels without incorporating metabolic context, or depend on insulin-derived IR indices that are limited by variability and restricted applicability in diabetic populations. The integration of eGDR may provide a more robust and clinically meaningful assessment. In this study, we utilized data from the 2013-2016 cycles of the National Health and Nutrition Examination Survey (NHANES) to investigate the association between eGDR and serum testosterone levels in U.S. adult men. We hypothesized that lower eGDR, indicative of greater IR, is associated with lower testosterone levels. By leveraging a large, nationally representative dataset, this study aims to provide novel epidemiological evidence regarding the metabolic correlates of TD and offer potential implications for metabolic health and male reproductive function.

## Methods

### Study design and participants

The NHANES program employs a rigorous sampling methodology to select a nationally representative cohort of the US population, conducting biennial assessments of health and nutritional status. The data clearly represent the nutritional and health status of the noninstitutionalized civilian population in the US because multistage sample weights are assigned to each survey participant. The study protocol was approved by the National Center for Health Statistics Ethics Review Board, and all participants provided written informed consent prior to enrollment. Therefore, no additional ethical approval was required for this analysis.

**Figure 1:**
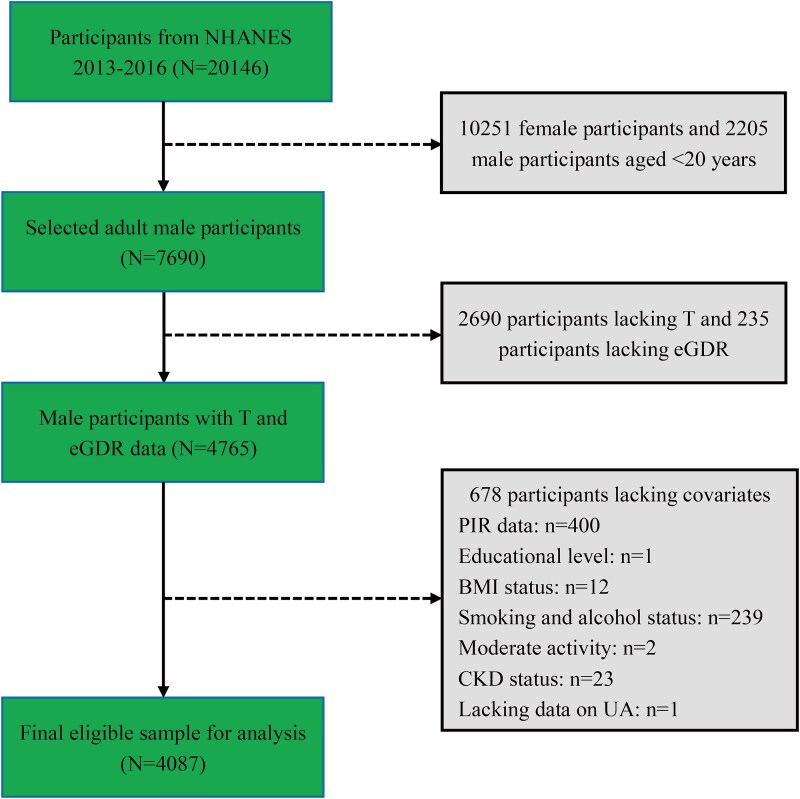
Flowchart of study participant selection process from NHANES 2013-2016.

This study utilized data from the 2013-2016 NHANES cycles, initially including 20 146 individuals. Exclusion criteria were applied as follows: (1) female participants (*n* = 10 251) and individuals younger than 20 years (*n* = 2205); (2) missing testosterone data (*n* = 2690) or unavailable eGDR values (*n* = 235); (3) incomplete covariate information (*n* = 678). After implementing these criteria, 4087 male participants were included in the final analysis. The participant selection process was shown in Figure 1.

### Exposure variables

The estimated glucose disposal rate (eGDR), expressed in mg/kg/min, was calculated using a validated formula derived from previous literature: eGDR = 21.158 − (0.09 × WC (cm)) − (3.407 × HTN (yes = 1, no = 0)) − (0.551 × HbA1c).^22^^,^^23^ Waist circumference was measured to the nearest centimeter (cm) using a standardized protocol. A horizontal line was drawn from the outermost point of the right iliac crest, extending to the right midaxillary line. The measuring tape was precisely positioned at the intersection of these marks to ensure accuracy. HTN was determined based on medical history, a systolic blood pressure of ≥140 mmHg, a diastolic blood pressure of ≥90 mmHg, or the use of antihypertensive medication during the survey. HbA1c levels were quantified using boronate-affinity chromatography combined with high-performance liquid chromatography. HbA1c was reported as a percentage of total hemoglobin, serving as a key indicator of long-term glucose metabolism.

### Outcome variables

Participants underwent overnight fasting before serum testosterone measurement to minimize potential metabolic influences. To account for diurnal variations, the CDC collected blood samples between 8:30 am and 11:30 am. Serum testosterone levels were determined using isotope dilution liquid chromatography–tandem mass spectrometry (ID-LC–MS/MS), a highly precise method that separates testosterone from binding proteins and removes interfering substances before analysis. According to the American Urological Association guidelines, TD was defined as total testosterone (TT) <300 ng/dL.^5^

### Covariates

We included potential confounding factors that might affect the association between eGDR and TT levels.^24^ Sociodemographic and lifestyle information was collected using standardized questionnaires, including age, body mass index (BMI), race/ethnicity (non-Hispanic White, non-Hispanic Black, Mexican American, other Hispanic, and other races), education level (<high school, high school graduate or equivalent, >high school), poverty-income ratio (PIR) (<1.3, 1.3-3.5, ≥3.5), marital status (single or cohabiting), smoking status (never, former, current), alcohol consumption (yes or no), and physical activity levels. Clinical biomarkers were measured following standardized laboratory protocols, including total cholesterol (TC), uric acid (UA), and high-density lipoprotein (HDL) cholesterol. Medical comorbidities included DM, CVD, hyperlipidemia, and chronic kidney disease (CKD).

Smoking status was categorized based on participants’ lifetime smoking history and current smoking behavior: never (answered “no” to both having smoked at least 100 cigarettes and currently smoking), former (answered “yes” to having smoked ≥100 cigarettes but “no” to currently smoking), and current (answered “yes” to both). Alcohol consumption was classified as yes (≥1 drink per week) or no (<1 drink per week). Physical activity was categorized based on participation in moderate activity (MA) or vigorous activity (VA). Detailed definitions of medical conditions are provided in Table S1.

### Statistical analysis

All analyses were conducted using the recommended sample weights according to the NHANES survey design and CDC guidelines to account for the complex sampling methodology. Descriptive statistics were used to summarize the data, with continuous variables presented as means ± SEs and categorical variables as weighted percentages. Group comparisons were performed using survey-weighted linear regression analysis for continuous variables and weighted chi-square tests for categorical variables. To evaluate the association between eGDR and TT levels as well as the risk of TD, we performed survey-weighted multivariable-adjusted linear and logistic regression analyses. The results of the linear regression were expressed as β coefficients (95% CI), while the logistic regression results were presented as odds ratios (ORs) with 95% CIs. eGDR was analyzed both as a continuous variable and as a quartile-based categorical variable. We constructed three models to progressively adjust for potential confounders: Model 1: Adjusted for eGDR only. Model 2: Further adjusted for key demographic factors, including age, race, marital status, education level, PIR, and BMI; and Model 3 further adjusted for lifestyle factors, including smoking status, alcohol intake, physical activity (moderate and vigorous), and medical history of DM, hyperlipidemia, CVD, and CKD.

A smoothing spline curve fitting approach was applied to explore the potential nonlinear relationships between eGDR and testosterone-related outcomes. For predictive analysis, we conducted receiver operating characteristic (ROC) curve analysis to assess the discriminatory power of eGDR in identifying TD, reporting the area under the curve (AUC) with 95% CI. Differences in AUC values between models were compared using the *Z*-test. For the ROC analysis, the optimal cut-off value for each immune cell-to-HDL-C ratio was determined by maximizing the Youden Index (sensitivity + specificity − 1), which identifies the point on the ROC curve with the best balance between sensitivity and specificity. Additionally, subgroup analyses were performed to determine whether the association between eGDR, TT levels, and TD varied by key demographic and clinical factors, including age, BMI, smoking status, VA, DM, hyperlipidemia, CVD, and CKD history. All statistical tests were two-sided, with *P* < .05 considered statistically significant. Statistical analyses were performed using EmpowerStats (www.empowerstats.com; X&Y Solutions, Inc., Boston, MA) and R version 4.0.5 (http://www.R-project.org, The R Foundation).

## Results

### Baseline characteristics

This study included 4087 male participants, with 1077 (26.4%) diagnosed with TD (TD). Baseline characteristics stratified by TD status are presented in Table 1. Participants with TD were generally older (49.32 ± 0.74 vs 45.92 ± 0.46 years, *P* = .002) and lower eGDR (6.23 ± 0.13 vs 7.94 ± 0.07, *P* < .0001). Metabolic disorders were more prevalent in the TD group, including HTN (48.51% vs 35.54%, *P* < .0001), DM (23.85% vs 12.92%, *P* < .0001), CVD (12.45% vs 7.57%, *P* < .001), hyperlipidemia (78.34% vs 63.66%, *P* < .0001), and CKD (17.15% vs 11.20%, *P* < .0001). Lifestyle differences were also observed, with TD participants less likely to engage in vigorous physical activity (24.81% vs 33.69%, *P* = .003). In addition, there were significant differences between the two groups in BMI, WC, HbA1c, UA, HDL levels, and smoking and drinking status (*P* < .001).

**Table 1:** Baseline characteristics of study participants with or without TD, weighted.

**Characteristics**	**Total participants**	**Participants without TD**	**Participants with TD**	***P*-value**
Participants number	4087	3010	1077	
Age, years	46.78 ± 0.35	45.92 ± 0.46	49.32 ± 0.74	.002
PIR	3.09 ± 0.08	3.10 ± 0.09	3.07 ± 0.08	.7
BMI, kg/m^2^	29.01 ± 0.15	27.88 ± 0.14	32.30 ± 0.31	<.0001
WC, cm	102.42 ± 0.42	99.31 ± 0.42	111.49 ± 0.79	<.0001
HbA1c, %	5.66 ± 0.02	5.57 ± 0.03	5.89 ± 0.04	<.0001
UA, mg/dL	6.06 ± 0.03	5.94 ± 0.03	6.42 ± 0.06	<.0001
TC, mg/dL	188.01 ± 1.08	187.57 ± 1.13	189.29 ± 1.87	.37
HDL, mg/dL	48.23 ± 0.44	49.89 ± 0.55	43.36 ± 0.50	<.0001
eGDR, ml/min/1.73m^2^	7.50 ± 0.07	7.94 ± 0.07	6.23 ± 0.13	<.0001
Total testosterone, ng/dL	420.40 ± 4.30	486.04 ± 3.67	228.50 ± 2.24	<.0001
Age group, %				.01
20-40y	37.51	39.67	31.19	
40-60y	37.30	36.43	39.86	
≥60y	25.19	23.90	28.95	
BMI, %				<.0001
Normal (<25 kg/m^2^)	25.16	30.11	10.67	
Overweight (25-30 kg/m^2^)	38.12	40.24	31.91	
Obese (≥30 kg/m^2^)	36.73	29.65	57.42	
PIR, %				.91
< 1.3	20.08	19.89	20.64	
1.3-3.5	35.11	35.23	34.76	
>=3.5	44.81	44.88	44.60	
Race, %				.23
Mexican American	9.16	8.99	9.67	
Non-Hispanic White	67.75	67.53	68.38	
Non-Hispanic Black	9.53	10.06	7.99	
Other Hispanic	5.53	5.43	5.82	
Other Race	8.03	7.99	8.15	
Marital status, %				<.001
Solitude	32.20	34.31	26.05	
Cohabitation	67.80	65.69	73.95	
Education, %				.97
Less than high school	14.14	14.18	14.01	
High school	22.74	22.65	22.97	
More than high school	63.12	63.16	63.01	
Smoke, %				<.0001
Never	29.47	26.58	37.94	
Former	20.26	22.06	14.99	
Current	50.27	51.37	47.07	
Alcohol, %				<.001
No	22.14	20.21	27.77	
Yes	77.86	79.79	72.23	
Vigorous activity, %				.003
No	68.57	66.31	75.19	
Yes	31.43	33.69	24.81	
Moderate activity, %				.14
No	53.05	52.03	56.01	
Yes	46.95	47.97	43.99	
Hypertension, %				<.0001
No	61.15	64.46	51.49	
Yes	38.85	35.54	48.51	
DM, %				<.0001
No	74.59	76.99	67.60	
Borderline	9.70	10.10	8.55	
Yes	15.70	12.92	23.85	
CVD, %				<.001
No	91.18	92.43	87.55	
Yes	8.82	7.57	12.45	
Hyperlipidemia, %				<.0001
No	32.60	36.34	21.66	
Yes	67.40	63.66	78.34	
CKD, %				<.0001
No	87.28	88.80	82.85	
Yes	12.72	11.20	17.15	

Abbreviations: BMI, body mass index; CKD, chronic kidney disease; CVD, cardiovascular disease; DM, diabetes mellitus; eGDR, estimated glucose disposal rate; HDL, high-density lipoprotein; PIR, poverty-income ratio; TC, total cholesterol; TD, testosterone deficiency; UA, uric acid; WC, waist circumference.

*Statistical Analysis*: Continuous variables are presented as mean ± SEs, and the categorical variables are presented as weighted percentages. Group comparisons were made using weighted linear regression analysis for continuous variables and weighted chi-square tests for categorical variables, respectively. A *P*-value of less than 0.05 was considered statistically significant.

### Association between eGDR and TT, and TD

Table 2 showed the results of survey-weighted linear and logistic regression analyses examining the association between eGDR, TT levels, and TD risk. In the unadjusted Model 1, higher eGDR was strongly associated with increased TT levels (*β* = 21.67, 95% CI, 19.45-23.89, *P* < .0001). After adjusting for demographic factors in Model 2, the association remained significant, though attenuated (*β* = 11.58, 95% CI, 7.62-15.55, *P* < .0001). In the fully adjusted Model 3, which accounted for lifestyle and clinical comorbidities, the association strengthened (*β* = 31.83, 95% CI, 22.13-41.54, *P* < .001). Quartile analysis showed that participants in the highest eGDR quartile (Q4) had significantly higher TT levels than those in the lowest quartile (Q1) across all models, with Q4 in Model 3 showing the largest effect size (*β* = 147.27, 95% CI, 66.99-227.55, *P* = .02). A positive trend across quartiles was observed (*P* for trend = .0001).

**Table 2:** Linear and logistic regression analyses for the association of continuous and quartile eGDR with TT and risk of TD, weighted.

	**Model 1**	**Model 2**	**Model 3**
**Total testosterone (ng/dL)-β (95% CI) *P*-value**			
Continuous eGDR	21.67 (19.45, 23.89), <.0001	11.58 (7.62, 15.55), <.0001	31.83 (22.13, 41.54), <.001
Q1	Reference	Reference	Reference
Q2	56.91 (37.18, 76.64), <.0001	25.00 (1.65, 48.35), .04	40.92 (−8.45, 90.29), .07
Q3	56.11 (35.53, 76.69), <.0001	32.59 (8.66, 56.51), .01	86.54 (24.87, 148.21), .03
Q4	170.59 (154.68, 186.49), <.0001	89.75 (60.89, 118.60), <.0001	147.27 (66.99, 227.55), .02
*P* for trend	<.0001	<.0001	.0001
**Testosterone deficiency-OR (95% CI) *P*-value**			
Continuous eGDR	0.79 (0.76, 0.82), <.0001	0.86 (0.82, 0.91), <.0001	0.68 (0.58, 0.80), .002
Q1	Reference	Reference	Reference
Q2	0.54 (0.41, 0.70), <.0001	0.74 (0.51, 1.07), .10	0.62 (0.26, 1.47), .14
Q3	0.42 (0.32, 0.55), <.0001	0.56 (0.40, 0.78), .002	0.32 (0.10, 1.01), .05
Q4	0.16 (0.12, 0.22), <.0001	0.36 (0.23, 0.58), <.001	0.20 (0.06, 0.70), .03
*P* for trend	<.0001	<.001	.005

Abbreviations: β, beta; BMI, body mass index; CI, confidence interval; CKD, chronic kidney disease; CVD, cardiovascular disease; DM, diabetes mellitus; eGDR, estimated glucose disposal rate; HDL, high-density lipoprotein; OR, odds ratio; PIR, poverty-income ratio; TC, total cholesterol; TD, testosterone deficiency; UA, uric acid; WC, waist circumference.

*Statistical Analysis*: Model 1: No variable adjustment. Model 2: Adjusted for age, race, marital status, education, PIR, and BMI. Model 3: Further adjusted for TC, HDL, UA, smoking status, alcohol consumption, VA, MA, history of DM, hyperlipidemia, CVD, and CKD.

In the fully adjusted Model 3, the association strengthened, with each unit increase in eGDR corresponding to 32% lower odds of TD (OR = 0.68, 95% CI, 0.58-0.80, *P* = .002). Quartile analysis showed that participants in Q4 had the lowest risk of TD compared to Q1, with the strongest effect observed in Model 3 (OR = 0.20, 95% CI, 0.06-0.70, *P* = .03). A clear inverse trend was seen across quartiles (*P* for trend = 0.005).

**Figure 2:**
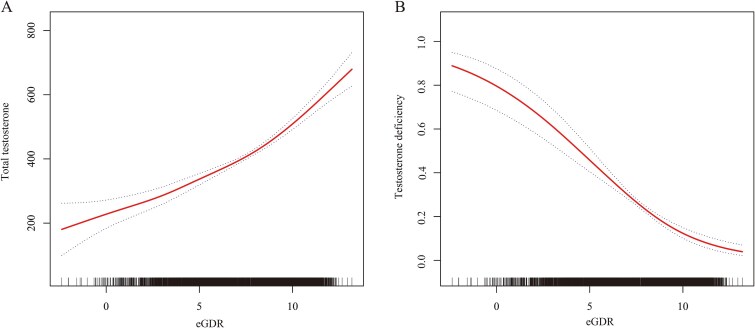
Graphics of smooth curve fittings between the eGDR and (A) TT and (B) TD.

As shown in Figure 2, the results of the smoothing spline curve fitting indicate a significant linear relationship between eGDR, TT, and TD. With increasing eGDR, TT levels gradually rise, while the risk of TD decreases significantly.

In summary, our findings consistently show that higher eGDR levels are associated with higher TT concentrations and a lower likelihood of TD in adult men. These associations remained robust across multiple regression models and were confirmed through both continuous and categorical analyses.

### Subgroup and ROC analyses

The subgroup analysis in Table 3 confirmed a consistent positive association between eGDR and TT levels across most groups. The subgroup analysis in Table 3 showed a significant positive association between eGDR and TT levels across most subgroups, including age, BMI, smoking status, VA, hyperlipidemia, and CKD. The association was strongest in 40-60 years (*β* = 34.47, 95% CI, 18.95-49.99, *P* = .002) and remained significant in 20-40 years (*β* = 32.61, 95% CI, 19.31-45.90, *P* = .001) and > 60 years (*β* = 28.22, 95% CI, 2.02-54.42, *P* = .04). It was also notable in VA (*β* = 44.88, 95% CI, 29.25-60.51, *P* < .001), hyperlipidemia (*β* = 26.34, 95% CI, 13.66-39.02, *P* = .003), and CKD (*β* = 40.50, 95% CI, 21.62-59.38, *P* = .003). However, the association was not significant in borderline DM (*P* = .12) and CVD (*P* = .07). The strongest interaction effect was observed for DM (*P* for interaction = .001), suggesting that glucose metabolism disturbances may influence the relationship between eGDR, TT levels, and TD risk. No interactions were observed in other subgroups (*P* for interaction >.05).

**Table 3:** Subgroup analysis on the association of continuous eGDR with TT, weighted.

**Subgroup**	***β* (95% CI)**	***P*-value**	***P* for interaction**
Age group			.22
20-40y	32.61 (19.31, 45.90)	.001	
40-60y	34.47 (18.95, 49.99)	.002	
>60y	28.22 (2.02, 54.42)	.04	
BMI			.07
Normal	51.13 (16.00, 86.26)	.01	
Overweight	37.71 (23.25, 52.17)	<.001	
Obese	28.77 (16.22, 41.31)	.001	
Smoking status			.50
Never	31.12 (19.19, 43.06)	<.001	
Former	37.12 (21.68, 52.56)	.001	
Current	27.25 (10.15, 44.35)	.01	
Vigorous activity			.18
No	27.43 (17.29, 37.57)	<.001	
Yes	44.88 (29.25, 60.51)	<.001	
DM			.001
No	35.15 (22.02, 48.27)	<.001	
Borderline	18.52 (-6.25, 43.30)	.12	
Yes	29.39 (14.94, 43.84)	.003	
Hyperlipidemia			.41
No	42.18 (30.31, 54.04)	<.001	
Yes	26.34 (13.66, 39.02)	.003	
CVD			.42
No	33.14 (24.31, 41.97)	<.001	
Yes	24.18 (-3.23, 51.60)	.07	
CKD			.49
No	29.72 (19.57, 39.88)	<.001	
Yes	40.5 (21.62, 59.38)	.003	

Abbreviations: TT, Total testosterone; PIR, poverty-income ratio; BMI, body mass index; WC, waist circumference; UA, uric acid; TC, total cholesterol; HDL, high-density lipoprotein; eGDR, estimated glucose disposal rate; DM, diabetes mellitus; CVD, cardiovascular disease; CKD, chronic kidney disease; β, beta; CI, confidence interval.

*Statistical Analysis*: All analyses were adjusted for all variables included in Model 3, except for the stratifying variable. The variables adjusted for included age, race, marital status, education, PIR, BMI, TC, HDL, UA, smoking status, alcohol consumption, VA, MA, history of DM, hyperlipidemia, CVD, and CKD.

**Figure 3:**
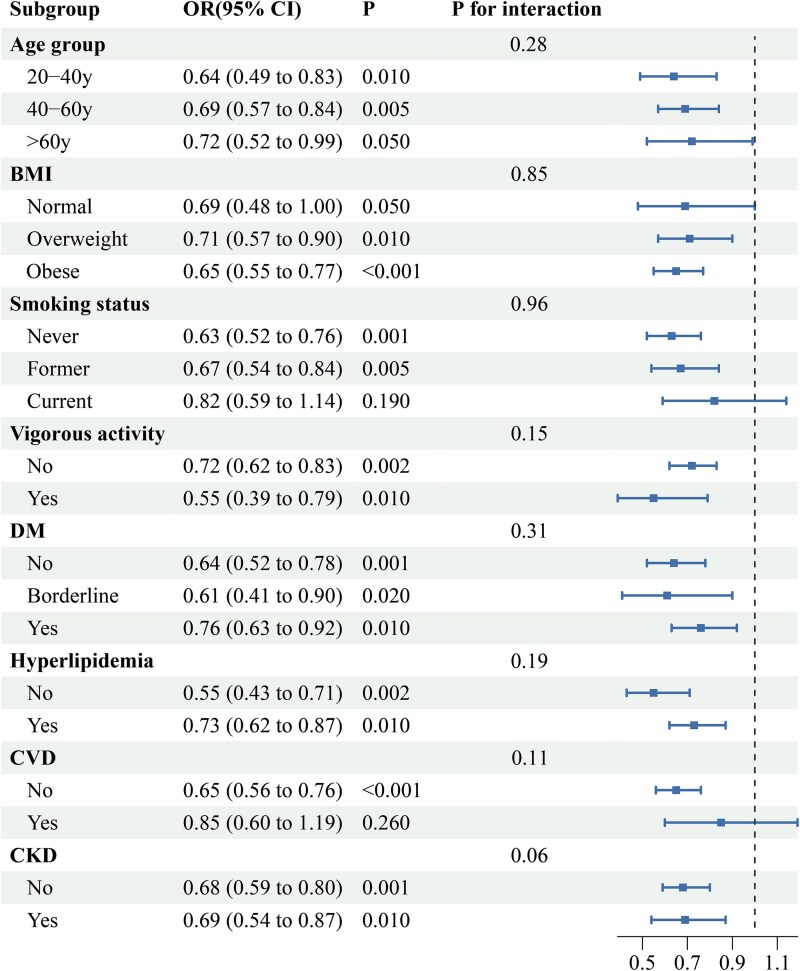
Subgroup analysis on the association of continuous eGDR with risk of TD, weighted. All analyses were adjusted for all variables included in Model 3, except for the stratifying variable.

The subgroup analysis in Figure 3 further confirmed the inverse association between eGDR and TD risk. The effect was strongest in 20-40 years (OR = 0.64, 95% CI, 0.49-0.83, *P* = .010) and 40-60 years (OR = 0.69, 95% CI, 0.57-0.84, *P* = .005), while slightly weaker in >60 years (OR = 0.72, 95% CI, 0.52-0.99, *P* = .050). The inverse association was also evident in obese individuals (OR = 0.65, 95% CI, 0.55-0.77, *P* < .001), those with VA (OR = 0.55, 95% CI, 0.39-0.79, *P* = .010), DM (OR = 0.76, 95% CI, 0.63-0.92, *P* = .001), and non-DM (OR = 0.64, 95% CI, 0.52-0.78, *P* = .001). The association remained significant in hyperlipidemia (OR = 0.73, 95% CI, 0.62-0.87, *P* = .010) and CKD (OR = 0.69, 95% CI, 0.54-0.87, *P* = .010) but was weaker in CVD patients (OR = 0.85, 95% CI, 0.60-1.19, *P* = .260) and current smokers (OR = 0.82, 95% CI, 0.59-1.14, *P* = .190).

**Figure 4:**
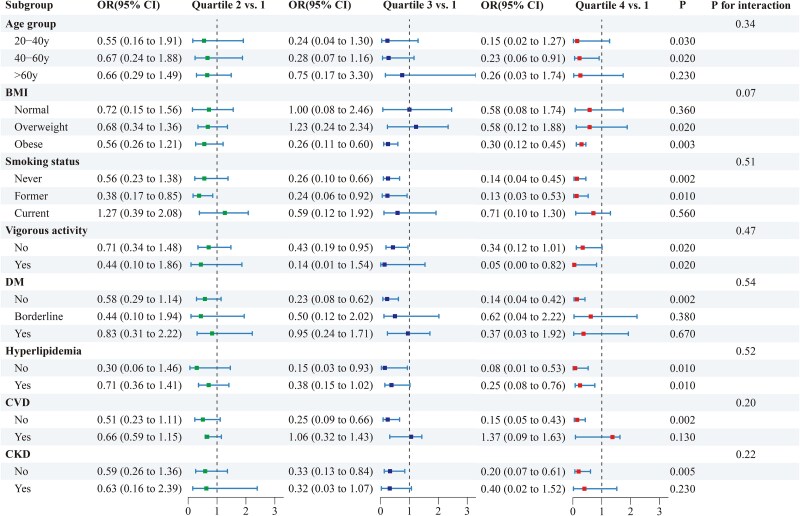
Subgroup analysis on the association of quartile eGDR with risk of TD, weighted. All analyses were adjusted for all variables included in Model 3, except for the stratifying variable.

To further enhance data visualization, Table 4 and Figure 4 showed the subgroup analysis of eGDR as a quartile-based variable in relation to TT levels and TD risk, which aligns closely with the findings from the continuous variable analysis. Across most subgroups, higher eGDR quartiles were associated with increased TT levels and a lower risk of TD, reinforcing the robust relationship between insulin sensitivity and testosterone regulation.

**Table 4:** Subgroup analysis on the association of the eGDR quartile with TT, weighted.

**Subgroup**	**Quartile 1**	**Quartile 2**	**Quartile 3**	**Quartile 4**	***P* for trend**	***P* for interaction**
Age group						.28
20-40y	Reference	23.75 (−24.34, 71.83)	66.35 (−1.26, 133.97)	115.38 (20.47, 210.29)	.01	
40-60y	Reference	59.96 (−0.62, 120.53)	128.68 (38.90, 218.47)	175.61 (63.70, 287.51)	.005	
>60y	Reference	45.17 (−14.92, 105.27)	65.42 (−35.31, 166.15)	175.18 (36.21, 314.15)	.01	
BMI						.28
Normal	Reference	65.5 (−58.42, 189.43)	75.46 (−77.69, 228.62)	108.75(−67.22, 284.72)	.17	
Overweight	Reference	38.31 (−3.38, 79.99)	41.43 (−48.76, 131.62)	104.95 (4.31, 205.59)	<.001	
Obese	Reference	45.03 (−5.03, 95.10)	103.43 (52.60, 154.25)	206.14 (35.89, 376.40)	<.001	
Smoking status						.65
Never	Reference	44.73 (−10.41, 99.87)	79.92 (23.36, 136.48)	139.6 (66.20, 213.01)	.001	
Former	Reference	52.81 (−9.43, 115.06)	96.9 (17.36, 176.45)	189.81 (66.30, 313.32)	.004	
Current	Reference	19.53 (−55.71, 94.77)	89.74 (8.80, 170.67)	110.94 (−10.60, 232.47)	.04	
Vigorous activity						.84
No	Reference	31.37 (−13.24, 75.98)	67.24 (12.54, 121.94)	118.8 (43.82, 193.77)	.003	
Yes	Reference	58.08 (−4.56, 120.72)	137.56 (48.60, 226.53)	209.05 (106.02, 312.07)	.001	
DM						.19
No	Reference	41.56 (9.47, 73.65)	91.68 (49.67, 133.69)	151.92 (97.95, 205.89)	<.001	
Borderline	Reference	41.74 (−59.09, 142.57)	33.13 (−123.52, 189.78)	39.71 (−190.22, 269.64)	.63	
Yes	Reference	39.08 (−31.36, 109.53)	70.8 (−21.25, 162.85)	155.64 (25.39, 285.89)	.03	
Hyperlipidemia						.86
No	Reference	55.47 (−47.82, 158.75)	89.58 (−30.99, 210.14)	153.77 (6.61, 300.92)	.01	
Yes	Reference	36.35 (0.05, 72.65)	85.00 (28.55, 141.44)	145.38 (70.19, 220.58)	.002	
CVD						.35
No	Reference	48.43 (4.07, 92.79)	90.57 (41.12, 140.01)	155.74 (93.76, 217.71)	<.001	
Yes	Reference	−27.79 (−107.97, 52.39)	75.14 (−119.95, 270.23)	1.23 (−189.37, 191.83)	.63	
CKD						.95
No	Reference	34.88 (−4.12, 73.88)	76.38 (28.22, 124.55)	129.56 (67.16, 191.96)	<.001	
Yes	Reference	76.11 (−41.57, 193.78)	125.26 (−9.59, 260.10)	252.27 (−9.66, 514.21)	.03	

Abbreviations: β, beta; BMI, body mass index; CI, confidence interval; CKD, chronic kidney disease; CVD, cardiovascular disease; DM, diabetes mellitus; eGDR, estimated glucose disposal rate; HDL, high-density lipoprotein; PIR, poverty-income ratio; TC, total cholesterol; TT, total testosterone; UA, uric acid; WC, waist circumference.

*Statistical Analysis*: All analyses were adjusted for all variables included in Model 3, except for the stratifying variable. The variables adjusted for included age, race, marital status, education, PIR, BMI, TC, HDL, UA, smoking status, alcohol consumption, VA, MA, history of DM, hyperlipidemia, CVD, and CKD.

**Figure 5:**
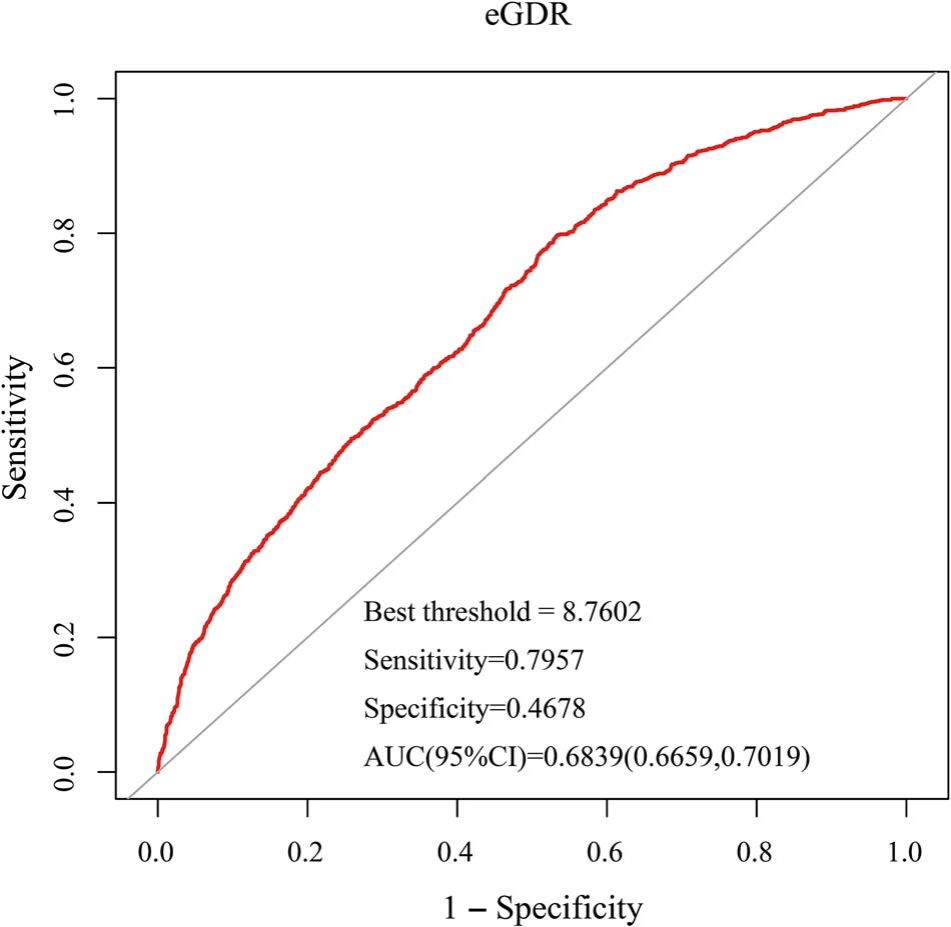
ROC curve analysis for eGDR in predicting testosterone deficiency. The analysis includes the area under the ROC curve (AUC) and the 95% CI.

As shown in Figure 5, the ROC analysis indicated that the AUC (95% CI) for eGDR in predicting TD was 0.6839 (0.6659, 0.7019). The optimal threshold for eGDR was 8.7602, with a sensitivity of 0.7957 and a specificity of 0.4678. These results suggest that eGDR has moderate discriminatory ability in identifying individuals at risk of TD, highlighting its potential role as a metabolic predictor of testosterone status in adult men.

## Discussion

In the present study, we investigated the association between eGDR and TT levels, as well as its relationship with TD risk, using data from NHANES 2013–2016. The key findings are as follows: (1) eGDR was significantly associated with both TT levels and TD risk, with higher eGDR corresponding to increased TT levels and a lower likelihood of TD. (2) A smoothing spline curve fitting approach revealed a linear relationship between eGDR and TT levels, with a gradual increase in TT as eGDR increased, whereas TD risk declined significantly with increasing eGDR, supporting the robustness of the association. (3) Subgroup analyses showed that these associations were consistent across most groups. (4) Receiver operating characteristic analysis indicated that eGDR had moderate predictive ability for TD (AUC = 0.6839, 95% CI, 0.6659-0.7019), highlighting its potential as a screening marker. Collectively, these findings emphasize the critical role of insulin sensitivity in testosterone regulation and suggest that eGDR may serve as a valuable metabolic marker for identifying individuals at risk of TD.

Insulin resistance is a key pathophysiological driver of metabolic syndrome and plays a crucial role in its progression through multiple mechanisms. It is also recognized as an independent predictor of TD or decline, with a well-established bidirectional relationship between IR and testosterone levels. Insulin resistance has been shown to reduce testosterone production, which in turn exacerbates obesity and further worsens IR.^25^ Tsai et al. conducted a study involving 221 middle-aged, nondiabetic men, demonstrating that testosterone levels were significantly and inversely correlated with insulin levels, HOMA-IR, and C-peptide in multivariable analysis.^26^ Our study extends this evidence by observing a strong association between eGDR, a novel IR marker, and both TT levels and TD risk. eGDR is a noninvasive and effective marker of IR that has been linked to complications in young T1D patients,^27^ such as retinopathy and nephropathy, as well as clinical outcomes in individuals with DM.^19^^,^^28^ In a large cohort of nondiabetic CKD patients, higher eGDR levels were associated with a reduced risk of CVD and mortality,^29^ while in both the general and nondiabetic populations, lower eGDR was independently linked to increased CVD risk and mortality.^28^^,^^30^^,^^31^ These findings underscore the utility of eGDR as a reliable tool for identifying high-risk individuals, independent of glucose tolerance status. Moreover, recent research has highlighted the potential of eGDR in identifying individuals at high risk for MetS and its complications, demonstrating a strong inverse correlation between eGDR and MetS prevalence.^21^ Given these previous findings, our study further supports the role of eGDR as a clinically relevant metabolic marker closely related to testosterone regulation and TD risk.

Although our findings demonstrate a strong association between eGDR and testosterone levels, the underlying mechanistic relationship remains uncertain. This may be attributed to several interrelated physiological factors. First, eGDR is a composite index incorporating blood pressure (HTN), glucose metabolism (HbA1c), and obesity (WC), all of which are known contributors to testosterone regulation. Insulin plays a role in stimulating gonadotropin-releasing hormone (GnRH) expression in the hypothalamus, thereby promoting GnRH secretion and regulating testosterone levels.^32^ However, hyperglycemia has been shown to suppress the expression of mitochondrial acetyl-CoA synthetase 3, leading to mitochondrial dysfunction and insulin receptor impairment in hypothalamic neurons. This cascade results in reduced GnRH gene and protein expression, ultimately suppressing GnRH neuronal activity and leading to testosterone decline.^33^ Second, aromatase activity in adipose tissue plays a critical role in testosterone metabolism. Aromatase, primarily expressed in adipocytes, converts testosterone into estradiol. Increased adipose tissue in obese individuals leads to elevated aromatase expression, which accelerates peripheral testosterone conversion and contributes to lower testosterone levels.^34^ Third, IR is linked to endothelial dysfunction, impairing vascular contraction and relaxation and promoting HTN.^35^^,^^36^ This process is primarily driven by reduced nitric oxide (NO) bioavailability and increased reactive oxygen species (ROS) production, both of which contribute to vascular dysfunction.^37^ Additionally, enhanced sympathetic nervous system activity and renin-mediated sodium retention further exacerbate vascular damage, promoting HTN. Obesity and IR are also associated with elevated circulating angiotensin II levels, further compounding endothelial dysfunction and reducing testosterone synthesis.^38^ Moreover, studies have shown that IR is associated with decreased vascular endothelial growth factor levels, a key mediator of vascular integrity. This highlights the complex interplay between metabolic dysregulation and vascular health, which may have downstream effects on Leydig cell function and testosterone production.^39^^,^^40^ Finally, obesity is a well-established risk factor for TD. In obese men, leptin resistance and inflammation-mediated mechanisms disrupt HPG axis function, ultimately leading to reduced testosterone secretion.^41^^,^^42^

The subgroup analysis demonstrated a consistent association between eGDR, TT levels, and TD risk across most subgroups, reinforcing the robustness of our findings. The positive relationship between higher eGDR and increased TT levels, as well as the inverse association between eGDR and TD risk, remained significant across age groups, BMI categories, smoking status, VA, DM, hyperlipidemia, CVD, and CKD status. Notably, a significant interaction was observed for DM status (*P* for interaction = .001), indicating that the relationship between eGDR and testosterone levels may be modified by glucose metabolism disturbances. While the association remained significant in both non-DM and DM individuals, the effect was somewhat attenuated in DM individuals, suggesting that long-term metabolic dysfunction, IR, and systemic inflammation may progressively weaken the beneficial impact of insulin sensitivity on testosterone production. These findings highlight the crucial role of insulin sensitivity in testosterone regulation, while also suggesting that diabetes may partially modify this relationship. Future research should explore the mechanisms underlying this interaction and assess whether improving insulin sensitivity through metabolic interventions could mitigate testosterone decline, particularly in DM populations. Interestingly, in some subgroups—such as individuals with CVD and current smokers—the association between eGDR and testosterone levels was not statistically significant. This may reflect smaller sample sizes, increased biological variability, or the influence of confounding factors such as chronic inflammation, medication use, or vascular impairment that affect testosterone levels through nonmetabolic pathways. Additionally, smoking has been associated with both increased and decreased testosterone in different studies, suggesting a complex, context-dependent effect that may dilute the metabolic association observed in other groups.

Although the ROC analysis demonstrated that eGDR had a statistically significant association with TD risk (AUC = 0.6839), this represents only moderate discriminatory power. Clinically, an AUC in this range suggests that while eGDR alone may not be suitable as a standalone diagnostic test, it can still serve as a useful screening indicator or an adjunct marker in conjunction with other clinical variables. Particularly in primary care or metabolic disease settings, where direct hormonal testing may be delayed or unavailable, eGDR offers a simple, noninsulin-based measure that could help flag individuals for further testosterone evaluation.

This study has several limitations that should be acknowledged. First, due to its cross-sectional design, we cannot establish causality between eGDR and testosterone levels. While lower eGDR may contribute to TD, it is also plausible that low testosterone promotes IR. This bidirectional relationship warrants cautious interpretation, and longitudinal studies are needed to clarify causality. Second, the diagnosis of TD in our study was solely based on biochemical measurements of serum testosterone levels, without incorporating clinical symptoms of TD syndrome. This may limit the clinical relevance of our findings, as testosterone levels alone do not fully define symptomatic hypogonadism. Future prospective studies should incorporate both hormonal and symptom-based definitions of TD to validate the predictive value of eGDR and other metabolic biomarkers in identifying clinically significant TD. In addition, we excluded individuals with missing testosterone or covariate data, which may introduce selection bias and reduce generalizability. Future studies with repeated hormonal assessments and comprehensive clinical evaluation are needed to confirm these associations. Third, although we adjusted for various metabolic and lifestyle factors, residual confounding cannot be ruled out. Unmeasured variables such as dietary habits, physical activity intensity, medication use (eg, testosterone therapy), and genetic predisposition may influence the relationship between eGDR and testosterone levels. Our covariate selection was guided by previous research to balance model comprehensiveness and stability; however, future studies should incorporate these additional factors to enhance the understanding of this association. Lastly, we excluded individuals with missing eGDR or testosterone data, which may introduce selection bias and limit the generalizability of our findings, particularly among populations with incomplete medical records. Although we applied multiple imputation and complete case analysis to address missing data, these approaches may not entirely eliminate bias. Future research should focus on including more comprehensive datasets or exploring advanced imputation techniques to improve the external validity and robustness of the findings. We are currently exploring the feasibility of establishing a clinical cohort to further investigate these associations prospectively.

## Conclusion

In this study, we observed a significant association between eGDR and TT levels, as well as an inverse relationship between eGDR and TD risk in US adult men. These findings suggest that insulin sensitivity, as reflected by eGDR, may be linked to testosterone regulation, with potential variations across different metabolic subgroups. Given the role of metabolic dysfunction in testosterone regulation, eGDR may serve as a useful metabolic marker for identifying individuals at risk of TD. Although our study is cross-sectional and cannot establish causality, the observed associations between eGDR and testosterone outcomes may have important implications. Future prospective studies are needed to determine whether improving insulin sensitivity can help preserve or restore testosterone levels.

## Supplementary Material

Supplementary_materials_qfaf075_Table_S1

## Data Availability

The data used in this study are available on the NHANES website at https://www.cdc.gov/nchs/nhanes/.
