# Supplementary material for: Association between estimated glucose disposal rate and testosterone level in US adult men: insights from NHANES 2013-2016
Source: Sex Med. 2025 Sep 15;13(4):qfaf075. doi: 10.1093/sexmed/qfaf075 (PMC12448460; doi:10.1093/sexmed/qfaf075)
Supplement: Supplementary_materials_qfaf075_Table_S1 [file supplementary_materials_qfaf075_table_s1.docx]

**Supplement Table S1**

**Definition criteria for medical complication variables**

| **Variables** | **Classification** | **Definition** |
| --- | --- | --- |
| Cardiovascular diseases | Yes | Having a history of any of the following conditions: coronary heart disease, congestive heart failure, angina, heart attack, and stroke. |
|  | No | None of the above. |
| Diabetes | Yes | 1. Doctor told you have diabetes,  2. Glycohemoglobin (HbA1c) ≥ 6.5%,  3. Fasting glucose ≥200 mg/dl,  4. Random blood glucose ≥ 11.1 mmol/l,  5. Two-hour OGTT blood glucose ≥ 200 mg/dl,  6. Use of diabetes medication or insulin. |
|  | Borderline | 1. Fasting blood glucose levels between 100 mg/dl and 125 mg/dl (impaired fasting glucose, IFG)  2. 2-hour post OGTT levels between 140 mg/dl and 199 mg/dl (abnormal glucose tolerance, FPG). |
|  | No | None of the above. |
| Chronic kidney disease | Yes | An estimated glomerular filtration rate (eGFR) <60 mL/min per 1.73 m², |
|  | No | eGFR ≥ 60 mL/min per 1.73 m² |
| Hyperlipidemia | Yes | 1. Triglycerides (TG) ≥ 150mg/dl,  2. Total cholesterol (TC) ≥ 130mg/dl,  3. Low density lipoprotein (LDL) < 40 mg/dl  4. High density lipoprotein (HDL) < 40 mg/dl  5. Use lipid-lowering medication. |
|  | No | None of the above. |
